# Prevalence and determinants of aggressive behavior among adults with problematic substance use in Northwest Ethiopia: a cross-sectional survey

**DOI:** 10.1186/s12888-022-04053-4

**Published:** 2022-06-15

**Authors:** Getasew Legas, Habte Belete, Sintayehu Asnakew

**Affiliations:** 1 Department of Psychiatry, School of Medicine, College of Health Sciences, Debre Tabor University, Debre Tabor, Ethiopia; 2 Department of Psychiatry, College of Medicine and Health Sciences, Bahir Dar University, Bahir Dar, Ethiopia

**Keywords:** Aggression behavior, Problematic substance use, Low-income, Ethiopia

## Abstract

**Background:**

The recent WHO data reported that a high treatment gap for behavioral illnesses (70%) in low- and- middle-income countries and the mortality rate of aggressive behavior reaches up to 32.1 per 100,000 populations in the region. However, the magnitude of aggressive behavior is not well stated in resource-limited settings. Therefore, the aim of this study was to assess the prevalence and determinant factors of aggressive behavior among adults with problematic substance use in northwest Ethiopia.

**Methods:**

A community-based cross-sectional study was employed from January to March 2019. A multi-stage cluster sampling method was used to screen a total of 4028 adults for problematic substance use by using the Cutdown, Annoyed, Guilty, and Eye-opener questionnaire (CAGE AID). Finally, 838 participants were positive for problematic substance use and interviewed for aggressive behavior using a modified overt aggression scale. Multiple logistic regression analysis was used to show the adjusted odds ratios (AOR) and p-value < 0.05 considered statistically significant. A multilevel binary logistic regressions model was employed for the hierarchical structure of two-level data for the individual and woreda/district levels.

**Results:**

The prevalence of aggressive behavior was found to be 37.9% (301/795, 95% CI: 34.5, 41.3). Stressful life events (AOR = 2.209, 95 CI; 1.423, 3.429), family history of mental illness (AOR = 4.038, 95 CI; 2.046, 7.971), comorbid physical illness (AOR = 2.01, 95 CI; 1.332, 3.032) and depressive symptoms (AOR = 2.342, 95 CI; 1.686, 3.253) were associated with aggressive behavior among individual with problem substance use.

**Conclusion:**

Aggressive behavior was found to be high among problematic substance uses. An individual with problematic substance use is recommended to be screened by health extension workers for aggressive behavior at the community level.

## Background

The recent global burden of the diseases reported that mental, substance use, and neurological disorders accounted for 258 million disability-adjusted life years (DALYs). From this, substance use disorders accounted for 14% of DALYs [1]. Approximately 155 to 250 million people (3.5% to 5.7% of the global population) had used illicit drugs at least once in the past year and 16 to 38 million people are estimated problematic drug users [2, 3]. The common risk factor of aggressive behavior in problematic substance use is an impairment of judgment due to substance intoxication [4, 5]. According to the World Health Organization report, in the modern emergency health care service, up 45% of substance emergency cases are presented with alcohol-related behavioral problems [6].

Aggressive behavior is a serious risk for mortality and morbidity and can include hostility, impulsivity, violence, and irritability [7]. Aggressive or violent behavior is responsible for 1.43 million deaths worldwide. The mortality rate of violent behavior is reached up to 32.1 per 100,000 populations in low- and middle-income countries [8, 9]. Problematic substance uses such as alcohol use had often the risk of aggressive behavior [10–12], 20–50% of alcohol-dependents had an incidence of violent behavior. Even alcohol abuse or dependency is more risk for aggressive or violent behavior [13–18].

In the USA, 35% of committed violence have occurred under the influence of drugs [19] while problematic substance use was responsible for up to 92% of domestic violence. Especially, stimulants are a promoter of violent behavior by reducing impulse control [20]. Intoxication and withdrawal symptoms of various substances can induce aggressive behavior with or without comorbid mental illness [21, 22].

The prevalence of aggressive behavior among problematic substance use varied across the globe. The prevalence of aggressive behavior among adults with problematic substance use ranged from 2.41% in India [23] to 95% in Malaysia [24]. The other study also showed that the prevalence of aggressive behavior among problematic substance users in India ranged from 35.2% [25] to 73% [26]. And the higher prevalence of aggressive behavior was reported, 32.5% in Iran [27], 62% in the UK [28], and 39.68% in Spain [29].

In Africa, the magnitude of aggressive behavior was reported from 27.6% in Nigeria[30] to 65.5% in South Africa [31]. A number of factors were reported as a risk for aggressive behavior in problematic substance users across different studies. Accordingly, male sex [32], stressful life events [33–35], low level of perceived social support [36, 37], family maladjustment [33, 37], depressive symptoms [38], family history of substance abuse [26], history of abuse [32, 38], family history of mental illness [26], and comorbid physical illness were significantly associated with aggressive behavior [26].

In Ethiopia, the prevalence of aggressive behavior among schizophrenia and bipolar patients was 26.6% and 29.4% respectively [39, 40]. But there is no study is conducted to assess the magnitude of aggressive behavior in problematic substance use. However, in the Ethiopian university, 54.3% of the students were reported with committing at least one act of physical violence or aggression [35]. Certain substance uses such as khat use, a natural stimulant with amphetamine-like effects, and alcohol use were found perceived causes of domestic violence, the perpetration of physical and sexual abuse through increasing anger and aggression [35, 41–43].

Even though the link between problematic substance use and aggressive behavior has been well addressed in developed countries, this issue needs to be addressed in resource-limited countries like Ethiopia. In this regard, there might be a disparity across high-and-low-income countries regarding the link between problematic substance use and aggressive behavior as the culture, lifestyle, type of habit, and behavioral responses to negative conditions could vary in the two zones. Hence, assessing and showing aggressive behavior among problematic substance users is important to enforce policymakers and different stakeholders to integrate mental health services with health care centers. Although the ultimate psychopathology of aggressive behavior poses a brain neuropsychology base, the primary risk factors could be a broad bio-psycho-social dimension which would be affected by socioeconomic factors, culture, community attitude, personal traits, and environmental conditions. Therefore, assessing the magnitude of aggressive behavior in problematic substance uses in resource-limited countries is compulsory to provide valuable evidence, and to inspire other future research on this area. To the best of researchers’ deep review, no research has been conducted in Ethiopia to address the magnitude of aggressive behavior among people with problematic substance use at the community level, where is the use of many substances such as alcohol drinking, khat chewing, and smoking is lawful. Therefore, this study was primarily intended to determine the prevalence and factors associated with aggressive behavior among problematic substance use in community residents of south Gondar zone, Northwest Ethiopia.

## Methods

### Study design, period, and setting

A community-based, cross-sectional study was employed between January to March 2019. The study was carried out in the South Gondar zone, Northwest, Ethiopia. The town is 99 km far from Bahir Dar (the capital city of the Amhara region) and 667 km far from Addis Ababa (the capital city of Ethiopia). Based on the 2007 census report; the total population size of South Gondar is estimated at 2,051,738. Alcohol, cannabis, nicotine (smoking), and khat are the most common available substances in the study area. All except cannabis are lawful in the community. In the study area, there is only one referral hospital that gave mental health services for problematic substance use and psychiatric disorders.

### Study participants

All adults who were living in the South Gondar zone were the source population. Study participants whose age was ≥ 18 years old and who lived for at least 6 months in the selected kebeles (the smallest administrative unit) were included in the study. On the other hand, adults who were unable to communicate due to severe illness were excluded.

### Sample size determination

The sample size has been calculated using Epi Info version 7 considering the following assumptions: the prevalence of aggressive behavior among problematic substance uses, 50% (as there was no previous study in the area); confidence limit, 5%; confidence level, 95%; design effect, 2; and non-response rate, 10%. Thus, a total sample size of 838 was obtained.

### Sampling procedures

A two-stage cluster sampling technique was utilized. First, we selected 4 woredas from the total of 15 woredas in the South Gondar Zone. Again, we randomly selected three kebeles in each of the selected 4 woredas. There were a total of 11,200 households in the selected kebeles. Then, screened all adults in each household for problematic substance use by using the CAGE AID questionnaire (Cut down, Annoyed, Guilty, and Eye-opener) until we get the calculated sample size of 838 adults with problematic substance uses. Based on the CAGE AID questionnaire, if a participant scores a minimum of two positive answers from a total of four questions, we considered problematic substance uses. We have screened 4028 adults for problematic substance use to get the calculated sample size of 838. Of those, 43 individuals have not assessed due to the reason 14 individuals were unable to communicate (complaining of illness), 15 individuals terminate their interview, and the other 14 individuals were not voluntary for further interview. Finally, we assessed aggressive behavior among 795 residents who were positive for problematic substance use.

### Method of screening for problematic substance uses

Participants were asked in a separated and secured room whether they have problematic substance uses or not. A pretested, interviewer-administered, anonymous, semi-structured, and standardized CAGE AID questionnaire was adapted. The data were collected by five mental health professionals and the questionnaire was translated into Amharic language (local working language). The training was given to the data collectors on the data collection tools and sampling techniques.

### Measurements

A 4 item of the CAGE AID questionnaire was used to screen problematic substance uses among community residents. Scoring of two or more positive answers from a total of four questions (yes/no) was considered problematic substance uses. Each item of questions was rated on a “yes” or “no” response which is valued at one point (1) and zero (0). CAGE-AID is an important tool to assess for other problematic drug uses (khat, tobacco, and cannabis) in addition to screening problematic alcohol uses in the community, and also has been utilized by previous Ethiopian studies [44, 45].

Aggressive behavior among individuals with problematic substance use was assessed by using the modified overt aggression scale [46]. This tool has a total of 16 items with four categories that measure four types of aggressive behaviors, namely “verbal aggression”, “aggression against property”, “auto-aggression”, and “physical aggression. The weighted value for every four subscales is different and the total score is 40. The verbal aggression subscale has a weight of × 1; which means, any score on this subscale should be multiplied by 1. The subscale against property has a weight of × 2; the auto-aggression subscale has × 3, while the physical aggression subscale has a weight of × 4. Aggression is expressed on five Likert scales which reflect the severity of the aggressive behavior (severity scores ranging from 0 (no aggression) to 4 points (maximum violence) for each category. A scoring of 3 or more (out of 40) was defined as having aggressive behavior [46] and the tool was validated in Nigeria [47]. The Cronbach’s alpha for the tool was 0.84.

The depressive symptom was measured using the patient health questionnaire (PHQ-9). PHQ-9 has nine items and each item has rated on a four-point scale, 0 (not at all), 1 (several days), 2 (more than half the days), and 3 (nearly every day) with the total score ranging from 0 to 27. Then a score of five or more on the PHQ-9 questionnaire indicates the presence of depressive symptoms for the last two weeks. PHQ-9 was validated in Ethiopia with the sensitivity and specificity of 86% and 67%, respectively [48]. Stressful life events were measured using a 12-item of List of Threatening Experiences(LTE), and the presence of stressful life events was explained by experiencing one or more stressful life events in the last 6 months [49].

Social support was assessed using a three-item Oslo social support scale which has three items with a range of between three and fourteen. From a total score of 14, scoring off 12–14 was categorized as strong support; a score of 9–11 was categorized as moderate social support; and a score of 3–8 was categorized as poor social support [50]. Perceived stigma among adults with problematic substance use was assessed using the Perceived Stigma Substance Abuse Scale. The instrument has 8 items with four Likert scales from strongly disagree to strongly agree. The total score ranges from 8 to 32 and individuals with higher scores from the mean indicate having greater perceived stigma [51].

A family history of mental illness was assessed by asking the participants whether there is a known family member who had a diagnosis of a mental illness or not. Clinical variables such as comorbid chronic medical illnesses and previous history of aggressive behavior were assessed by asking the participant if they had any diagnosed medical, surgical, or neurological illness and aggressive behavior before the study.

Socioeconomic factors such as participants’ level of education were categorized as uneducated (who were unable to read and write), and educated (who were able to read and write). Their living circumstance was checked if they were living with their family or alone, and their job status had also checked if they had a job or not.

### Data processing and analysis

The data were entered into Epi data version 3.1 and analyzed by using Statistical Package for Social Science (SPSS) version 20. We present the result frequencies/percentages, odds ratios, and adjusted odds ratios were calculated using logistic regression. A multilevel binary logistic regression model was constructed to account for the two-level data for the individual and the woreda/district level.

## Results

### Socio-demographic Characteristics

A total of 795 respondents were interviewed with a response rate of 94.8%. The majority of the respondents 606 (76.2%) were male while 711 (89.4%) were Orthodox Christian by religion. More than half, 460 (57.9%) study participants were jobless. Regarding ethnicity, 751(94.5%) were Amhara. 644(81%) of study subjects was educated. Most of the respondents 512(64.4%) were currently not married and 643(80.9%) of the study subjects were residing in urban areas (Table 1).

**Table 1:** Socio-demographic characteristics respondents in south Gondar zone, northwest Ethiopia, 2019 (*n* = 795)

Variable	Category	Frequency	Percentage
Age	18–40	481	60.5%
	> = 40	314	39.5%
Sex	Male	606	76.2%
	Female	189	23.8%
Ethnicity	Amhara	751	94.5%
	other	44	5.5%
Educational status	Non-educated	151	19%
	Educated	644	81%
Religion	Orthodox	711	89.4%
	Muslim	84	10.6%
Marital status	Currently not married	512	64.4%
	Married	283	35.6%
Living circumstance	With family	475	59.7%
	Alone	320	40.3%
Residence	Rural	152	19.1%
	Urban	643	80.9%
Job status	Jobless Has job	460 335	57.9% 42.1%

### Psychosocial and clinical factors

Of the total 795 participants, three hundred thirty-six (42.3%) had poor social support, three hundred seventy (46.5%) had perceived stigma due to their problematic substance use and one hundred six (13.3%) were experienced at least one stressful life event. Regarding clinical factors; one hundred twenty-eight (16.1%) of participants had a comorbid chronic physical illness, and two hundred ninety-one (36.6%) had depressive symptoms. Forty-nine (6.2%) of individuals had a family history of mental illness and seven hundred (88.1%) of problematic substance users hadn’t a previous history of aggressive behavior (Table 2).

**Table 2:** Distribution of psychosocial factors among respondents in south Gondar zone Northwest, Ethiopia, 2019 (*n* = 795)

Variable	Category	Frequency	Percentage
Social support	Poor	336	42.3%
	Moderate	286	36.0%
	Strong	173	21.7%
Previous history aggression behavior	Yes	95	11.9%
	No	700	88.1%
Stigma	Yes	370	46.5%
	No	425	53.5%
Stressful life events	Yes	106	13.3%
	No	689	86.7%
Comorbid physical illness	Yes	128	16.1%
	No	667	83.9%
Depressive symptoms	Yes	291	36.6%
	No	504	63.4%
Family history of mental illness	Yes	49	6.2%
	No	746	93.8%

### Prevalence of Aggressive Behavior

Aggressive behavior in problematic substance use was 301/795, 37.9% (95% CI: 34.5, 41.3). Regarding the domains of aggressive behavior; verbal aggression was the highest domain eighty (26.6%) and auto aggression was the lowest fifty-five (18.3%). Physical aggression was seventy-three (24.3%) while aggression against property was sixty-two (20.6%).

### Determinant Factors of Aggressive Behavior

In this study, the previous history of aggression behavior, co-morbid physical illness, depressive symptoms, stressful life events, and family history of mental illness were associated with aggressive behavior on bivariate analysis. While, co-morbid physical illness, depressive symptoms, stressful life events, and family history of mental illness were found to be significantly associated with aggressive behavior among adults with problematic substance use on multivariable analysis (Table 3).

**Table 3:** Bivariate and multivariate analysis of aggressive behavior among problematic substance use in south Gondar zone, northwest Ethiopia 2019

Variables	Category	Aggressive behavior		COR 95% CI	AOR 95% CI	*P* -value
		**Yes**	**No**			
Comorbid physical illness	Yes	72	56	2.46 (1.68, 3.61)	2.07(1.37, 3.11) *	0.001
	No	229	438	1	1	
Previous history of aggressive behavior	Yes	46	49	1.64 (1.06, 2.52)	1.56(0.99, 2.46)	0.56
	No	255	445	1	1	
Stressful life event	Yes	58	48	2.218(1.46, 3.35)	2.09(1.36, 3.23) *	0.0001
	No	243	446	1	1	
Depressive symptoms	Yes	143	148	2.12(1.52, 2.85)	2.16(1.58, 2.94) *	0.0001
	No	158	346	1	1	
Family history of mental illness	Yes	35	14	4.51(2.38, 8.54)	3.94 (2.02, 7.69) *	0.0001
	No	266	480	1	1	

^*^ *p* < 0.05, COR = crude odds ratio; AOR = adjusted odds ratio; 1 = reference group

### Multilevel analysis

The results of the multilevel binary logistic regression analyses are reported in Table 4. This examination showed that aggressive behavior was not varied significantly across woredas /districts (β = 0.082, *p* = 0.231). Model 1 presents the effects of individual-level of variables. For the individual-level variables; comorbid physical illness, family history of mental illness, depressive symptoms, and stressful life events were associated with aggressive behavior (Table 4).

**Table 4:** Multilevel logistic regression analyses of contributing factors to aggressive behavior among participants

**Model 1 **Fixed effects β				
**Model variables**	Β	*P*	**OR**	**95% CI**
Intercepts	.082	.231		
Comorbid physical illness	.692	.001	2.01	1.332–3.032
Depressive symptoms	.851	< .001	2.342	1.686–3.253
Family history of mental illness	1.396	< .001	4.038	2.046–7.971
Stressful life events	.792	< .001	2.209	1.423–3.429
Model χ2 =	1.138	*P* < .001		

## Discussion

Aggressive behavior has a major impact on mortality through homicide or suicide and is the main reason for emergency admission among many psychiatric patients. In the current human psychological problems, aggressive behavior is the primary cause of many conflicts, injuries, and crimes, and recent studies showed that up to 35% of emergency department visits are directly or indirectly substance-related problems [52]. In this regard, substance misuse is responsible for leading to aggressive behavior which could increase morbidity and mortality that cause imprisonment, and limits social interaction in the community.

The prevalence of aggressive behavior in this study was 37.9% (95% CI: 34.5, 41.3). The current study was similar to a study done in India which was 35.3% [25] and 39.7% in Spain [29]. However, the magnitude of aggressive behavior in the current study was lower than in studies done in Malaysia 95% [24], the UK 62% [28], and South Africa 65.5% [31]. But, the finding was higher than studies conducted in Iran 32.5% [27] and Nigeria 27.6% [30]. The variation might be due to the number of participants in the study. That is, a study was done in the UK, only 86 individuals were included, in Nigeria 298, Iran 280, Malaysia 200, and in South Africa, 84 study participants were included. The other possible reason might be the difference in the assessment tool; used Ahvaz Aggression Questionnaire (AAQ), Malaysia Aggression Questionnaires (AQ) scale, and India used Buss and Perry Aggression Questionnaire. But, we utilized the overt modified aggression scale. The other possible reason for this discrepancy might be cultural differences.

Regarding the associated factors, we found an association between depressive symptoms and aggressive behavior. The finding of this study was similar to studies done in the USA and Ethiopia [38, 53]. Moreover, irritability or impulsivity is frequently observed in people with depressive symptoms and correlates positively with aggressive behavior [54–56].

Our study also identified family history of mental illness had a statistically significant positive correlation with aggressive behavior. The finding has been supported by studies done in India and the USA [26, 38]. In this regard, the effect of genetic predisposition has been explained as biological predisposing factors [57, 58], and evidence explains that a family history of behavioral illness has been associated with a higher rate of impulsive and aggressive behavior [59].

We found an association between comorbid physical illness and aggressive behavior. The finding was supported by other studies [60, 61]. The possible reason might be medical illness and neurological disorders can increase the risks of anger or aggressive behavior [56, 62]. The dual effect of their behavioral problems and the burden of physical illness might worsen and lead to dissatisfaction in their life which might be exposed to aggressive behavior.

The odds of developing aggressive behavior were found higher among participants who experienced stressful life events than their counterparts. The finding was supported by previous studies [34, 63]. Experience of stressful life events has a strong link with amplification of aggressive behavior [64] or exposure to stressful life events has associated with reactive aggression [63], and high stress in young people has a direct impact on the development of substance use and aggressive behavior [65]. Even some people who have low activity of catechol-O-methyltransferase and monoamine oxidase A genes are more sensitive to stressful life events that exhibited higher levels of aggressive behavior [66].

### Limitations of the study

Interpretation of our findings is hindered by many limitations, including the cross-sectional design, which impedes causal inferences. The other limitation is female participants were small which may hamper the representativeness of our findings.

## Conclusion

Aggressive behavior among problematic substance users was found to be high. Having comorbid depression, a family history of mental illness, comorbid medical/surgical illness, stressful life events, and poor social support were the significant predictors of aggressive behavior. Therefore, in addition to control of amendable factors, regular screening of aggressive behavior in problematic substance uses at the community level is recommended.

## Data Availability

All relevant data are available in the manuscript.
